# Supplementary material for: Are carbon emissions trading and green financial instruments synergistic? -Comprehensive quantitative research based on content analysis
Source: PLoS One. 2024 Mar 7;19(3):e0298601. doi: 10.1371/journal.pone.0298601 (PMC10919601; doi:10.1371/journal.pone.0298601)
Supplement: S1 Data — (ZIP) [file pone.0298601.s001.zip › Data/地级市Z_F工作报告.zip!/║╙▒▒/╒┼╝╥┐┌╩╨/└┤╫╘╣1⁄2╓┌║┼í╛┬φ┐╦╩2╛▌═°í┐_2017.docx]

各位代表：

现在，我代表张家口市人民政府向大会报告政府工作，请予审议，并请市政协各位委员提出意见。

张家口市第十三届人民政府任期是从2013年4月开始的。四年来，在习近平总书记系列重要讲话精神指引下，在省委、省政府和市委的坚强领导下，面对 “三期叠加”、经济下行压力加大的挑战，全市人民牢固树立新发展理念，团结奋斗， 攻坚克难， 经济社会各项事业实现了平稳有序发展。地区生产总值年均增长6.5%，2016年完成1461亿元，人均生产总值突破3万元。 一般公共预算收入年均增长7.4%，2016年完成141.7亿元。 财政支出年均增长12.5%，2016年达到415.2亿元。固定资产投资年均增长8.8%，2016年完成1631.3亿元。 特别是在党中央、 国务院的亲切关怀下， 我们在奋力拼搏中迎来了京津冀协同发展、 举办冬奥会、可再生能源示范区三大历史机遇，张家口由此进入了绿色崛起、跨越发展的新阶段!

四年来，冬奥会申办取得成功，筹办工作进展顺利。在习近平总书记亲自关怀下，省委、省政府大力推动，我市与北京市密切配合，高标准完成各项工作，申奥一举成功，赢得了为国增光、绿色崛起的重大历史机遇。目前，冬奥系列规划编制、 核心区征地拆迁、 基础设施和配套建设有序推进。成功举办了2016-2017国际雪联自由式滑雪雪上技巧世界杯总决赛。2021年自由式滑雪和单板滑雪世锦赛申办成功。奥运经济开始起步，同多个国家、多个企业建立了友好合作关系，法国冰雪装备龙头企业MND公司落户我市。

四年来，生态建设不断加强，环境质量持续改善。累计造林530万亩，2016年完成230万亩，投资和规模均创历史新高。森林覆盖率从2012年的33.6%提高到39%，入选国家生态文明先行示范区和山水林田湖生态保护修复试点城市。建成国家级自然保护区1个、 森林公园2个、 湿地公园6个。 发展及改善节水灌溉面积113.6万亩，高效节水推广率达到28%。 全市8处国省控河流断面均达到国家地表水Ⅲ类水质标准。 综合施策， 加强大气污染防治，空气质量持续向好，2016年空气质量综合指数 4.50，PM2.5年均浓度下降到32μg/ m3， 优良天数达到288天，保持了京津冀地区最好水平。

四年来， 新兴产业日益壮大， 产业结构得到优化。确立了 “大生态、 大旅游、大数据、 大健康、 新能源、新技术、 高端制造” 新兴产业体系， 北方硅谷、 吉利沃尔沃、 张北云基地、 旗帜乳业等一批重点项目顺利推进。高新技术产业占规模以上工业增加值的比重从2012年的11.1%提升到25.1%。 服务业发展进一步提速， 占经济总量的比重从2012年的41%提高到44.6%。 2016年， 旅游接待游客达到5194万人次， 总收入519亿元， 分别增长35%和46%。 www.libaiwu.com 政府工作报告。退出炼钢产能389万吨、 炼铁产能495万吨、水泥产能140万吨、煤炭产能880万吨。规模以上工业增加值年均增长5.3%，2016年完成373.9亿元。高耗能传统产业占规模以上工业增加值比重从2012年的59%下降至40%。单位生产总值能耗年均下降4.6%。 可再生能源示范区获得国务院批准， 全市风光发电装机容量达到1057万千瓦， 为建设 “低碳奥运专区” 和 “低碳城市” 奠定了基础， 同时也肩负起了为国家能源革命探索新模式的重任。

四年来， 协同发展不断深入， 改革创新成效明显。以习近平同志为核心的党中央审时度势、 高瞻远瞩， 开启了京津冀协同发展鸿篇巨制， 张家口积极融入其中，受益日多。 我市与北京市签署了科技、 文化、 人才、 产业等7个方面合作协议， 与北京市8个区建立了对口帮扶机制。 医疗、 教育合作不断深入， 生态、 交通、 产业协同实现了新突破。 京张高铁、 延崇高速等重大基础设施开工建设， 洋河新区、 高铁沿线协同创新平台建设开始起步。北汽福田智能制造基地建设加快，京张国企多家合资公司注册完成。累计引进北京资金1047亿元。2016年引进项目117个，到位资金385.2亿元。在协同协作中，大力推动金融创新，组建张家口金融控股集团，完成21家投资类公司及合伙企业注册，上市企业增加到30家，2016年新增13家。2016年底贷款余额2026.4亿元， 增长20.6%。 搭建资本平台，通过基金引导、统贷统还，争取地方债券240亿元， 支持基础设施、 公益性项目建设和化解政府债务。2016年筹集资本金、偿债基金23亿元，争取政策性金融支持， 投放棚户区改造贷款192.2亿元、 美丽乡村贷款36.89亿元、植树造林贷款34.4亿元。

四年来， 脱贫攻坚扎实推进， “三农” 工作迈上新台阶。认真落实 “六个精准” “五个一批” “八大脱贫工程”。启动了4.9万人易地扶贫搬迁， 累计减少贫困人口82.7万人。承办了第二届全国乡村旅游与旅游扶贫工作推进大会和全省易地扶贫搬迁现场观摩调度会。培育壮大现代农业，加快一二三产业融合发展，市级以上农业产业化龙头企业发展到342家， 其中国家级龙头企业3家， 产业化经营率达到64%。持续深化农村改革，土地承包经营权确权登记颁证工作顺利推进。土地流转面积达到360万亩，市级以上现代农业示范园增加到43个，多种形式规模经营格局初步形成。

四年来，新型城镇化较快推进，城乡面貌明显提升。完成了行政区划调整， 主城区面积由868.96平方公里扩展到6377.81平方公里。基础设施建设提速， 张唐铁路、 张涿和张承高速公路通车，张大、张呼铁路建设加快，太行高速开工，高速公路通车里程达到927公里。新改建干线公路308公里、农村公路4977公里。宁远机场开通10条航线，通达13个城市。 “四城同创”扎实开展，公共秩序及环境卫生进一步改善，浅山区过度开发得到控制，违规建设治理成效明显。 棚户区改造完成6.6万套。 县城建设步伐加快， 张北、怀来被评为国家园林县城，涿鹿、万全等8个县区被评为省级园林县城。1043个村庄的基础设施条件得到改善，建成市级美丽乡村235个，省级美丽乡村88个。 城乡面貌和公共服务进一步提升，全市常住人口城镇化率从2012年的47.5%提高到54.2%。

四年来， 各项社会事业较快发展， 人民生活进一步改善。 财政用于民生支出年均增长14.1%。 城乡居民可支配收入年均增长10.6%和13.5%， 2016年分别达到26069元和9241元。 多渠道拓宽就业岗位， 城镇累计增加就业21.14万人， 高校毕业生就业3.09万人， 农村劳动力转移就业15.66万人。 完善社会保障体系， 低保线和扶贫线实现 “两线合一”。 城乡居民基本医疗保险完成整合， 大病保险实现全覆盖。 全市建设农村示范性互助养老院120所， 社会力量兴办养老机构83家。 建设各类保障性住房9.03万套， 改造农村危房13.5万户。 教育 “全面改薄”工程实现国家 “双过半” 要求， 改扩建幼儿园630所， 中心城区新建学校6所， 全市小学生实现了6周岁入学， 职业教育和特殊教育达到全国先进水平， 张家口教育学院升级为本科院校。 2016年高考本科一批上线率达到19.6%，比2012年提高了11.4个百分点。 县级公立医院综合改革不断深化， 市级公立医院综合改革开始实施，19家医院全部取消了药品加成。 文化事业实现新发展， 市 “三馆” 工程主体完工， 察哈尔都统署、 元中都等遗址保护项目全面完成， 中华三祖、 阳原泥河湾、 蔚县剪纸等文化品牌影响力进一步扩大。

四年来， “放管服” 改革持续推进， 政府自身建设不断加强。 深入开展党的群众路线教育实践活动、 “三严三实” 专题教育活动、 “两学一做” 学习教育， 认真落实中央 “八项规定” 精神， 坚决纠正 “四风”， 大力推进机关作风整顿， 各级政府机关作风和营商环境明显好转。 严格执行国务院 “约法三章”， 自觉接受人大法律监督、 政协民主监督和社会舆论监督， 切实加强行政监察和审计监督。 大力简政放权， 非行政许可审批事项全部取消。 市本级审批事项精简481项， 精简比例达71%。 实施了 “双随机、 一公开” 制度， 整合了公共资源交易平台， “多证合一、 一照一码” 登记制度全面实施， 市场主体增加到21.2万户。强化社会综合治理， 认真做好群众信访工作， 安全生产、食品药品安全、 社会治安形势总体稳定。 国防动员、 双拥共建、 民族宗教、 新闻广电出版、 外事侨务、 人民防空、档案史志、 气象地震、 援疆援藏、 妇女儿童、 老龄、 残疾人等工作都取得了新的成绩。

各位代表！回首走过的四年，有共同奋斗的艰辛，也有共同收获的喜悦。我们深刻地体会到，以习近平同志为核心的党中央的坚强领导是我们各项事业顺利前进的根本保证；历届班子的持续努力是我们各项事业顺利前进的坚实基础；全市人民和社会各界的大力支持是我们各项事业顺利前进的强大动力。成绩的取得，是省委、省政府和市委正确领导的结果，是全市上下团结奋斗的结果。在这里，我代表市十三届人民政府，向全市人民，向人大代表、政协委员，向各民主党派、 工商联、 无党派人士和人民团体， 向驻张解放军、武警官兵，向中省直驻张单位，向所有关心、支持张家口发展的各级领导、离退休老干部、各界人士和海内外朋友，致以崇高的敬意和衷心的感谢！

在总结成绩的同时，我们也清醒地看到我市经济社会发展中存在的问题和挑战。一是经济结构性矛盾比较突出。经济发展方式依然比较粗放，传统产业质量效益较低，新兴产业发展还不够快， 实体经济经营困难， 县域经济总体较弱，新旧动能转换压力巨大。二是生态环境还比较脆弱，综合治理任务艰巨，污染源点多面广，大气、水、土壤污染防治任务很重。全社会环保意识还不够强，生态文明建设长效机制亟待建立。三是城市规划、建设、管理水平不高，城市功能不完善，承载能力不强。交通秩序、环境卫生、物业管理水平低，市区和部分地区房价上涨快，群众意见比较大。四是农村基础设施落后，面貌差，生产生活方式粗放，组织程度低，新型城镇化和美丽乡村建设任务繁重。五是民生工作短板很多。 城乡居民收入提高还不够快， 就业、 医疗、 教育、文化、养老等公共服务需要大力度提升。扶贫开发进入了攻坚阶段， “硬骨头”很多，特困人群兜底需要新机制。六是改革力度还不够大。 政府职能转变不到位， 行政效率不高，一些政府部门和工作人员担当意识、服务意识不强，庸懒散浮现象时有发生。营商环境不够好，市场主体不够活跃。七是经济社会一些领域发展不够规范，建章立制任务很重。我们必须直面问题，迎难而上，潜心研究，集思广益，综合施策，扎实奋斗。

春节前夕， 习近平总书记冒着严寒， 亲临张家口看望困难群众， 视察冬奥会筹办工作， 发表了重要讲话， 为我们进一步指明了方向， 极大地鼓舞了广大干部群众。 我们必须强化政治意识、 大局意识、 核心意识、 看齐意识， 以习近平总书记系列重要讲话和重要指示精神引领工作航向， 指导全部工作。 我们必须倍加珍惜机遇， 竭力奋发有为， 不辜负总书记的亲切关怀和殷切期望。 我们必须深入贯彻 “创新、 协调、 绿色、 开放、 共享” 新发展理念， 深入贯彻省第九次党代会、 省十二届人大五次会议和市第十一次党代会精神， 围绕完成 “三大历史任务” 和实现 “两个圆满完成、 两个升级进位、 三个显著提升”， 走绿色发展、 生态强市之路， 锐意改革， 勇于担当， 加快转型升级、 跨越提升， 打造冀北绿色创新发展新高地， 建设宜居宜业宜游新家园， 努力交出冬奥会筹办和本地发展两份优异答卷。

今后五年， 要着力做好七个方面工作。

（一）聚精会神筹办冬奥。深入贯彻总书记关于扎扎实实推进冬奥会筹办的重要指示，精细落实 “四个办奥” “五个着力”要求，坚持世界眼光、国际标准、中国特色，带动脱贫攻坚、协同发展、新型城镇化、生态建设、转型升级取得重大突破。要在高标准规划上狠下功夫。充分体现中国元素、 地方特色， 把张家口赛区规划与京津冀协同发展规划、北京冬奥会整体规划有机融合， 使筹办工作在时间、 空间、要素上实现无缝对接， 做到协调、 有序、 平稳、 高效推进。要在高标准建设上狠下功夫。 坚持百年大计， 发扬工匠精神，严格按规划和计划推进，严格执行节能环保标准，全面完成场馆设施建设和服务功能配套任务，确保建成优质、生态、人文、廉洁的精品工程。要在因势发展奥运经济上狠下功夫。坚持民生优先、生态优先、产业优先，把绿色发展理念贯穿筹办冬奥会全过程，发展绿色高端产业，让冰雪运动和冰雪产业在张家口落地生根。 确保为世界奉献一届 “精彩、非凡、卓越”的冬奥盛会，走出一条绿色发展、生态强市的新道路！

（二）精准推进脱贫攻坚。深入贯彻总书记关于扎扎实实推进脱贫攻坚的重要指示，突出精准扶贫、精准脱贫这条主线， 统筹推进生产扶贫、 易地扶贫、 生态扶贫、 教育扶贫、社保兜底扶贫、医疗救助扶贫，实行 “绣花式” “滴灌式”扶贫，加大产业扶贫力度，为贫困群众培育可持续发展的产业、可持续脱贫的机制、可持续致富的动力。突出深度贫困地区这个重点， 加大力量倾斜、 资金倾斜、 政策倾斜，滴水穿石，聚力攻坚。突出脱贫质量这个关键，做到领导工作实、任务责任实、资金保障实、督查验收实，保证脱贫工作务实、脱贫过程扎实、脱贫结果真实。把扶贫同扶志结合起来，注重扶贫先扶智，着力激发贫困群众发展生产、脱贫致富的主动性， 着力培育贫困群众自力更生的意识和观念，鼓励支持贫困群众发奋图强、勤劳致富。通过各方不屈不挠的共同奋斗， 确保到2020年彻底甩掉贫困帽子， 和全国人民一道昂首进入全面小康!

（三）统筹协调优化生态。深入贯彻总书记关于生态文明建设的重要指示，协调推进山水林田湖综合治理，高标准建设首都水源涵养功能区和生态环境支撑区。标本兼治提升大气质量，全面加强污染源管控，加快推进可再生能源示范区建设，建立清洁能源供应体系，推进农村散煤替代。坚持工业污染、生活污染、农业污染一同防治，地表水、地下水统筹治理，上下游、干支流联合治污，推进土壤分类分级管理和污染治理。持续推进生态修复，多种模式造林绿化。全面开展地下水超采综合治理，大力推动节水和水资源循环利用。大幅提升生态环境质量，森林覆盖率达到50%以上，地下水采补基本平衡，主要河流水体水质全部达到地表水Ⅲ类以上标准，PM2.5控制到25μg/m3以内， 空气质量达到世卫组织第二阶段标准。让我们张家口的天更蓝，地更绿，水更清，风光更靓丽！

（四）创新发展加速转型。深入贯彻总书记关于创新发展的重要指示，坚持创新驱动，加速新旧动能转换。要扎扎实实推进去产能， 钢铁产能全部退出， 矿山退出80%以上，实现 “基本无矿市”。 注重提升传统产业， 大力发展 “四大两新一高” 主导产业， 加快培育新动能， 着力扶持实体经济，大力发展民营经济。壮大现代物流、信息服务、金融服务、电子商务、文化创意等现代服务业，建设国家全域旅游示范区，全面提升现代服务业发展水平。高新技术产业占规模以上工业增加值比重超过50%，服务业占生产总值比重达到60%以上。提高开发区建设水平，实现省级开发区县区全覆盖，确保建成1家以上国家级开发区。园区经济对全市发展的贡献率超过60%。 大力优化营商环境， 深化行政审批、商事制度改革，降低制度性交易成本，营造重商、亲商、养商、安商的良好氛围。大力推动大众创业、万众创新，让智慧和财富充分涌流。力争使我市综合经济实力进入全省中上游行列！

（五）深度融入协同发展。深入贯彻总书记关于京津冀协同发展的重要指示，把发展绿色经济、建设绿色城市和承接非首都功能疏解有机结合起来。要以京张携手筹办冬奥会为牵引，加强规划统筹，完善工作计划，细化工作方案，快速有序推进。突出重点领域，强力推进交通、环保、产业领域率先突破向纵深发展， 为协同发展开路架桥、 奠定基础。要大力加强协同创新平台建设，发挥高铁、高速沿线节点区域、 重点城区、 重点园区的比较优势， 加快建立协调统一、关联互动的财税金融、产权交易、科技研发、创业就业、教育医疗等方面的合作政策，形成共建共享、协作配套、互助共赢的新机制。 要注重资本合作， 引入更多战略合作伙伴，积极推进合作一级开发运营，形成百舸争流的协同发展新格局，早日把张家口建成京津冀，特别是北京机构、企业、人才的事业产业新基地、生产生活新家园！

(六) 补齐短板促进和谐。 深入贯彻总书记关于以人民为中心的重要指示，城乡统筹共进，硬件软件一起补，难点痛点一起抓，加快提升公共服务综合能力。要加强规划，加大投入，开放创新，加快教育、医疗事业发展，基本形成完善的教育体系和医疗卫生体系。大力发展文化体育事业，实施冬奥惠民工程。 要加强城乡建设管理， 加快文明城市创建，推进公共服务均等化，提高城区承载力。要加强社会建设，健全基本生活保障体系，织密扎牢民生保障网。大力促进就业创业，切实关爱老人、妇女、儿童、残疾人。持之以恒抓好安全生产、食品安全、防震减灾工作。要加强社会治安综合治理， 全方位提高人民群众安全感。 加强民族、 宗教、侨务和国防动员工作，营造安定的社会环境，凝聚起共创美好新生活的强大合力！

（七）深化改革激发活力。深入贯彻总书记关于深化改革的重要指示，完善体制机制，增强发展动力。要把深化农业农村改革与推进新型城镇化结合起来，坚持集约节约，统筹推动生态、产业共进共生。大力发展精品农业，打响张家口绿色品牌。要把深化国有企业改革、投融资改革与大力发展民营经济、扩大对外开放结合起来，鼓励优化重组，发展混合所有制，深度开放地方市场，广泛搭建资本平台，去杠杆，降成本，防风险，全面激发市场主体的创造力。要把深化行政管理体制改革与建章立制结合起来， 突出政令畅通、利企便民、生态环保、协同发展、提升管理服务、创建文明城市等重点，通过制定政府规章、提请人大立法，形成一系列制度政策规范，推动张家口成为秩序之城、活力之城、和谐之城！三、 2017 年重点工作

前景是美好的，任务是艰巨的。我们一定要认真学习领会、深入贯彻落实习近平总书记系列重要讲话、特别是视察张家口重要讲话和重要指示精神，以供给侧结构性改革为主线，坚持目标导向，突出问题导向，注重顶层设计，注重夯实基础，注重重点突破。加快推进冬奥筹办、生态建设、脱贫攻坚、转型升级、协同发展、可再生能源示范区、新型城镇化等各方面重点工作， 发展绿色经济， 建设绿色城市。2017年， 完成三大历史任务、 交出两份优异答卷要迈出关键步伐。

2017年的主要预期目标是： 全市地区生产总值增长8%， 规模以上工业增加值增长4%， 服务业增加值增长10%以上， 全部财政收入增长10%， 一般公共预算收入增长3% （可比增长9%）， 固定资产投资增长12%， 社会消费品零售总额增长10%， 实际利用外资增长7%， 万元生产总值能耗下降4%。PM2.5年均浓度控制在31μg/m3以内， 化学需氧量、 二氧化硫、 氨氮、 氮氧化物减排完成省下达目标任务。 城乡居民人均可支配收入均增长10%以上， 居民消费价格指数涨幅控制在3%左右， 城镇登记失业率控制在4.4%以内。

重点推进十个方面工作：

（一）扎扎实实推进冬奥会筹办工作。把筹办冬奥会当作头等大事，聚焦赛事核心，把握时间节奏，严格按规划和计划高标准推进各项工作。

加快规划落地和基础设施建设。认真落实 “四个调整”，如期完成崇礼区7项规划和冬奥系列规划。加强与北京冬奥组委沟通协调， 完成 “三场一村” 设计招标和施工图设计。对确定的76个与冬奥会直接相关的项目， 制定详细实施方案。强化土地管控和征收工作，确保列入今年开工计划的竞赛场馆、 宁远机场改扩建、 核心区道路等38个项目按时开工。其它项目加快前期工作，按时间节点推进。实施科技冬奥计划，把节能智能先进技术融入场馆基础设施建设。加强监督检查，确保 “廉洁办奥”。

加快冰雪运动推广和冬奥人才培养。成立冬季运动管理中心，统筹推动冬季运动发展。抓好国家和省级冰雪训练基地建设。以能够独立举办国际冰雪综合赛事为目标，创新投资、建设、运营机制，多层次、多形式建设冰雪场馆，市奥体中心完成基础工程。 组织好 “大好河山?激情张家口” 冰雪季系列活动， 继续推进 “冬令营” 以及冰雪运动进校园、进社区、进企业活动。深化与国内外体育组织、冰雪运动发达地区和专业院校的交流合作，加强河北北方学院、张家口学院、 市职教中心相关专业建设， 组建冬季运动专业学校。积极引进和培养赛事运营管理、技术保障、教练和运动员等专业人才。全面启动志愿者培养计划，加快全民冰雪知识和冰雪技能普及。

加快奥运经济发展。 大力引进一流企业和体育运营机构， 积极培育冰雪运动装备、 冰雪旅游和冰雪赛事新业态。抓好冰雪运动装备产业园建设， 年内法国MND项目投产。抓紧申报综合保税区，打造集研发、制造、服务、交易于一体的全国冬季体育用品生产交易基地。推进京张体育文化旅游产业带建设，推动冰雪运动与旅游、文化、科技、会展等产业融合发展。启动冬奥食品供应基地建设。注重在发展奥运经济中扩大就业，提升公共服务水平，让人民群众在筹办中有更多获得感。

加快文明素质提升。 大力开展全国文明城市创建活动，着力实施城市管理提升、 市场环境整治、 文明社区创建等八大攻坚行动， 坚持问题导向， 突出重点难点， 明确责任分工， 广泛动员群众参与， 大幅提高社会文明程度和公民文明素质， 打造城市文明的名片。 年内确保进入省级文明城市行列并获得全国文明城市提名资格。 以 “创城” 为统揽， 组织各行各业开展 “迎冬奥、 讲文明、 树新风” 系列活动。 制定文明行为标准化规范， 率先在机关、 企事业单位和旅游、 交通、 商贸、 餐饮、 娱乐等行业开展文明礼仪、英语口语及手语大培训。 扩大志愿者队伍， 规范出租车驾驶， 劝阻不文明行为， 倡导文明风尚。 让文明之花在冬奥之城遍地开放！

（二）扎扎实实推进脱贫攻坚。贯彻落实 “五个统筹推进” “六个精准”，年内确保9.7万贫困人口达到脱贫标准，同步巩固提升脱贫成果。

强化精准扶贫。 把生产扶贫作为主攻方向， 因地制宜发展扶贫产业， 做到户户有增收项目， 人人有脱贫门路。把易地扶贫搬迁作为重要补充， 推进产业园区和移民社区“两区同建”， 确保贫困群众搬得出、 稳得住、 能致富。 完成367个村4.9万人搬迁， 启动271个村4.78万人搬迁。把生态扶贫作为双赢之策， 设立生态基金， 加大生态投入，多上生态项目， 扩大生态就业， 增加生态收益。 把教育扶贫作为治本之计， 整合教育资源， 改善教育条件， 落实好有关政策， 为建档立卡贫困家庭的大学生和高中生每人提供5000元资助金， 确保基础教育全覆盖， 切断贫困 “代际传递”。 大力支持 “生态建设、 绿色项目、 城镇化” 三位一体区域开发， 鼓励股权合作， 促进村集体和村民多增收、长受益。 对康保、 沽源、 尚义、 张北、 阳原5个深度贫困县， 做好精准规划， 制定实施方案， 精细推进集中攻坚。

强化政策兜底。 加大困难人群救助力度。 突出抓好农村低保对象、 长期患病者、 孤寡老人、 残疾人、 五保户等重点群体的精准帮扶。 建立农村低保标准动态调整机制，抓好低保与扶贫两项政策无缝衔接， 既要保好， 又要兜住。农村低保标准提高到3400元。 改善贫困县区医疗卫生条件， 完善基本医保、 大病保险、 医疗救助三重保障机制。设立市、 县 （区） 两级特困救助基金， 创新助困机制， 确保兜底全覆盖。

强化脱贫考核。 把完善建档立卡动态管理机制、 脱贫致富路子创新、 脱贫质量提升、 脱贫成果巩固、 干部工作作风、 企业社会责任等进行综合考量。 引入第三方， 科学评估， 严格考核， 严明奖惩。 坚决杜绝数字脱贫、 被脱贫。

（三）扎扎实实推进生态建设。编制完成全域生态文明建设规划及实施方案，划定生态红线，分解工作任务，协调推进山水林田湖综合治理。

持续开展 “蓝天行动”。 全面落实 《张家口市大气质量提升规划实施方案 （2016-2022）》， 针对影响空气质量的主要因素，统筹城乡环境，盯住重点行业、重点地区和重点时段，突出散煤、建筑扬尘、机动车、排放不达标企业四大重点，精准施策、综合治理。完成燃煤锅炉淘汰任务。宜电则电，宜气则气，大力推进清洁能源替代，以中心城区、县城为重点，结合美丽乡村建设快速向中心镇、中心村扩展，逐步实现农村全域覆盖。 建立以电代煤专项基金， 今年启动2000万平方米以上清洁能源供热工程。 实施智慧环保， 完善企业排放监测体系，加强火电、水泥、化工等重点行业企业的常态监控，严格标准倒逼，杜绝死角死面。严禁直接焚烧秸秆和生活垃圾。开工建设全市生活垃圾一体化处理和秸秆综合利用工程。

持续开展 “绿地行动”。 宜乔则乔、 宜灌则灌、 宜草则草。大力推进工程化造林、项目化管理， 完成造林绿化240万亩，森林覆盖率达到42%。在冬奥核心区、八角台城市绿核、 高速公路和高铁两侧、 中心城区等重点区域和重点部位，高标准打造绿化精品工程。坚持政府主导和市场机制相结合，创新造林及管护机制，鼓励社会资本参与。实行第三方验收，严防弄虚作假。推进国家湿地公园建设，加快矿山生态修复。

持续开展 “碧水行动”。 严格水资源税征收和水资源管理。 深入推进坝上试点县水权确权登记和农业水权水价改革。继续开展地下水超采综合治理及农田水利设施建设，完成宣化洋河、蔚县壶流河两处大型灌区节水改造，新增及改善节水灌溉44万亩以上。治理水土流失面积200平方公里。积极开展人工增雨增雪， 加大污水处理和雨水收集利用力度，建设海绵城市。多种形式兴建蓄水设施，开工建设乌拉哈达水利枢纽工程。 认真贯彻 “水六十条”， 全面落实市县乡三级河长制。 全市河流Ⅲ类以上水质断面达到50%以上，城市饮用水源全面达到Ⅲ类水质， 地下水质级全面保持稳定。

持续开展 “净土行动”。 实施畜禽养殖废弃物处理和资源化利用、危险废物治理、涉重金属企业清理整顿、关停搬迁企业污染场地治理修复等专项整治。加强农业面源污染防治，全面使用优质农地膜，建立农地膜回收体系。加大有机肥料研发、生产、推广力度。规模化畜禽养殖场配套建设粪污处理设施比例达到100%。 开工建设6个无害化处理厂，实现病死畜禽无害化处理全覆盖。

（四）扎扎实实推进产业转型升级。深入推进供给侧结构性改革，聚力实体经济，创新发展模式，加快动能转换。

加速发展战略性新兴产业。 围绕发展 “四大两新一高”主导产业，把重大产业项目的引进和建设作为重点，大力引进和实施一批产业关联度高、带动能力强的重大产业支撑项目，抓好市级260个、县级400个重点项目，力争完成投资1000亿元以上。 加快实施 “大智移云” 引领计划， 促进信息化和工业化融合。快速推进吉利沃尔沃汽车城、上海张江分园、张北云计算产业园、北汽福田智能制造基地、冰雪运动装备产业园等龙头项目建设，做大做强产业集群。高新技术产业增加值增长25%以上。

加速发展现代服务业。坚持生产性服务业和生活性服务业并重，推进信息技术与服务业深度融合，推动商业模式创新、跨界融合。培育和引进一批教育医疗、康养休闲、交通物流、 商贸物业等领域的龙头企业。 积极发展 “夜经济”，增强城市经济活力。借势冬奥会，把旅游业与生态建设、美丽乡村、新型城镇化、相关产业融合起来，推动全域旅游发展。打破行政区划限制，统筹规划，整体包装，通过大力招商引资，引进专业化、实力强的大公司合理开发。以建设京张体育文化旅游产业带、 草原天路等为轴线， 依托张家口“春有百花秋有月， 夏有凉风冬有雪” 的自然禀赋， 融入传统文化元素和地方特色文化风格，打造特色旅游大区和精品景区， 创优全域旅游服务， 推进区域旅游一体化融合发展，形成以线连点、以点带面、点面结合的全域旅游格局，促进全市农业、工业和服务业全面快速发展。现代服务业增加值增长20%以上。

加速改造提升传统产业。 深入实施 “中国制造2025”战略。创新投入方式，大力支持企业科技研发，改进生产装备和工艺水平，推动制造业向高端迈进。扶持中煤机等一批“两化融合” 程度较高企业进行智能化改造。 引导企业强化质量意识，弘扬工匠精神，大力提高质量标准，持续实施品牌战略， 打造更多知名品牌， 培育一批 “百年企业”。 鼓励企业战略重组、横向联合。坚决去、主动调、加快转，压减炼钢产能216万吨、 煤炭产能178万吨， 退出矿山90个。支持宣钢发展新业态、加快转型。采取切实措施，引导金融资本更多投向实体经济，支持中小微企业发展。落实各项政策，加快民营经济发展。支持民营企业优化重组，进行规范的股份制改造，建立现代企业制度，加强诚信体系建设，加快转型升级，不断做大做强。关心爱护企业家，支持企业家专心创新创业。 加大 “个转企、 小升规、 规改股、 股上市”力度，新增规模以上工业企业60家以上。

加速释放创新创业活力。 引入战略伙伴， 支持大企业、 科研院校、 创新型中小微企业设立专业化 “众创空间”， 共建 “双创” 平台， 实现各县区 “大创园” 和 “众创空间” 全覆盖。 实施科技型中小企业 “双倍增” 和高新技术企业 “双提升” 计划， 大力培养科技 “小巨人”。 科技型中小企业达到1500家， 高新技术企业达到60家。 推动中小企业创业基地建设， 建立创业扶持奖励基金， 加大创业资本支持， 年内新登记市场主体6万户以上。 在充分利用好本土人才的同时， 采取更为有效的人才政策， 完善创新成果利益分配机制， 鼓励高端人才来我市创业发展。年内引进高层次人才团队5个以上、 海内外高层次人才30名以上。

加速推进农业供给侧结构性改革。建立县级土地流转中心， 鼓励规模经营。 坚持 “集约农业、 精品农业、 循环农业、节水农业”发展方向，强化科技支撑，调优、调绿、调新农业结构， 增加优质绿色农产品供给， 提高农业供给质量。整合创建知名农业品牌，发展现代农业产业园区，打造重要农产品生产保护区、特色农产品优势区和奥运农产品生产基地， 建设精品农产品展示销售中心和区域冷链物流中心。 积极推进 “互联网+现代农业”， 壮大农村电商、 休闲农业、 乡村旅游等新产业新业态。 支持旗帜、 雪川、 禾久、弘基、张农集团等农业龙头企业在推行农业标准化生产、与城镇化融合发展方面发挥示范带动作用。新增市级以上现代农业产业园区20个以上，新登记家庭农场2000家。

（五） 扎扎实实推进协同发展。 以京张高铁建设和北京对口帮扶为契机，把引进优质项目和疏解北京非首都功能有机结合起来， 政府顶层推动， 部门精准对接， 企业深度合作。

注重平台建设。做好协同创新平台建设规划，推进高铁沿线、洋河新区、北方硅谷、大数据基地、蔚县产业新城等重点区域平台建设， 注重引进教育、 医疗、 科技、 文化资源，加快配套设施建设，争取有关方面支持，制定保障激励政策，为高端产业规模化引入奠定良好基础。

注重主体撬动。 在深化与中信、 国家电网、 北京城建、海淀国投、河北国控、华夏幸福等大企业、大集团合作的同时，大力引进新战略伙伴，建设协同创新的集中承载地，吸引北京总部基地、研发机构和科技企业入驻。与北京结对的8个县区要率先突破，其它县区都要广开渠道。 协商选定合作企业，组成一级开发运营主体，做好中长期计划，制定年度行动方案，政府跟进，切实支持，形成政企积极互动、各方踊跃参与的生动局面，把协同发展推向一个新阶段。

注重精准对接。细化方案，分解责任，全面落实与北京的各项合作协议， 特别是对口帮扶合作 “五项重点任务”。认真制定协同发展工作计划，分工协作、有序推进。利用地近、人熟优势，找准目标，盯住跟进，积极与北京方面进行多渠道、多层次精准对接。建立常态化对接洽商机制。充分发挥京张互派挂职人员的作用，聚焦协同，无缝衔接，持续跟进，不断突破。

（六） 扎扎实实推进可再生能源示范区建设。 按照国务院批准的 “三大创新” “四大工程” “五大功能区” 规划，创新思路，细化方案，全面推进。

着力推动立体式开发。 把可再生能源开发与植树造林、 美丽乡村建设有机结合起来， 政府主导推动， 联合国有企业、 社会资本， 加大土地流转、 土地整理力度。 在协调推进生态修复、 中心村和中心镇建设的同时， 为可再生能源的基地化、 规模化开发留出空间。 在张北、 沽源、 康保、 尚义等建设一批多能互补示范项目， 在察北、 阳原、宣化等启动建设一批风光小镇， 尚义抽水蓄能电站项目完成前期工作。 年内全市新增可再生能源装机400万千瓦以上。

着力推动综合应用。 全面启动以电代煤、 以电代油工程， 加快清洁能源供应体系建设。 争取国家、 省政策支持，创新运营模式， 探索建立政府、 发电企业、 电网公司、 用户侧四方机制， 政府适当补贴、 电网大力支持， 大幅降低用户侧下网电价， 加快可再生能源就地消纳应用， 加快“低碳奥运专区” 和 “低碳城市” 建设。 建立微电网， 在民用、 工业、 交通、 农业、 建筑等方面实施一批示范项目。沽源风电制氢项目完工投产。 配合支持国家电网加快柔性直流、 特高压建设， 为可再生能源在京津冀及更大区域应用畅通输出通道。

着力延伸可再生能源产业链条。与国家电网、高校、科研机构组建可再生能源战略与技术研究院，加强技术研发、成果转化和战略发展研究。 加快可再生能源装备产业园建设，年内落地3家以上品牌企业。亿华通氢燃料电池、康保和宣化太阳能组件竣工投产。金风科技研发生产基地、涿鹿光伏组件、赤城智能光伏产品基地等项目开工建设。启动可再生能源科技创新城和综合商务区建设。

（七）扎扎实实推进新型城镇化。坚持以人为本，建设绿色城市。更加注重提高户籍人口城镇化率，更加注重城乡基本公共服务均等化，更加注重环境宜居和历史文脉传承，努力提升人民群众获得感和幸福感。常住人口城镇化率达到55.3%。

顶层设计规划引领。坚持世界眼光、国际标准、中国特色。实施新规划、构建大交通、拉开大框架、形成新格局。坚持全域规划，完善现代城镇体系。完成城市总规修编和分区详规，实行城市、国土、产业、交通等 “多规合一”。洋河新区、高铁新城等重要区域要用最先进理念和国际一流水准，编制经得起历史检验的规划，努力建设绿色、现代、智慧新城，打造优美生态环境，聚焦高端业态。在建筑设计上充分体现中华文化元素，在建设过程中精雕细琢，以工匠精神打造百年建筑， 留下千年传承。 抓住我市列入国家城市“双修”试点的机会，制定城市修补、生态修复的规划和实施方案。谋划启动城市轨道交通建设。

城乡统筹加快建设。在中心城区，新城区要上高品位，老城区要出新形象。实施南扩、北延、西拓、东优，加快建设速度。高铁综合枢纽及其周围区域按照城市新地标、中央商务区、经济增长极的定位，完善规划，开工建设。洋河新区完成基础设施配套，张家口学院、国家信息安全产业园、科教城、健康城开工建设。做好南站至北站既有铁路线路的轨道公共交通改造利用，加快推进市区到万全区、崇礼区公交的开通。新建经一路、经三路、沙岭子西站至城市干道的连接线、万经快速路等6条城区道路，打通纬二桥、北关大街、容辰大街、新辰路等23条城市断头路。启动城市更新，推进背街小巷、 老旧小区、 大杂院改造提升， 加快综合管廊、慢行系统、停车场、绿地、公厕、垃圾中转站、便民市场建设。在医院、学校等附近建设停车位2000个。在县域，实施县城建设三年攻坚行动，完善公共服务设施。坚持产城教融合， 在做大县城的同时， 大力撤并行政村， 建设中心村、中心镇。加强历史名镇名村、传统村落、历史建筑的保护开发，因地制宜打造有历史记忆、地域风格、各具特色的小城镇。今年重点打造左卫、洋河南等20个重点镇和30个特色风情小镇。在加快在建公路项目实施的同时，开工建设太行山、张北至崇礼、张北至尚义的高速公路，启动张北至康保高速公路建设。实施总投资20亿元的农村公路建设项目。年内全市完成棚户区改造5万套。

有力调控加强管理。坚持以政府为主提供基本保障，以市场为主满足多层次需求的思路，落实 “房子是用来住的，不是用来炒的”基本定位，制定促进房地产行业健康有序发展的具体措施。立足完善城市功能，合理增加商住用地，科学设定土地出让条件。建设平价房、限价房，保障本地居民和引进人才刚性需求，遏制房价过快上涨。加强市场监管整顿，严厉查处开发、销售、中介违规行为。深入推进违法建设专项治理，以 “零容忍”的态度杜绝新的违法建筑。改革城管体制，强化基层管理，着力解决群众反映强烈的难点、痛点。大力整顿交通秩序，优化交通道路系统，科学设置交通指示标识，建设智能交通。完善社区党支部、居委会、业主委员会和物业公司 “四位一体”社区管理运营机制，强化基层管理主体责任， 提升社区自治水平。 下大功夫解决停车、 环卫、 占道、 噪音等问题， 让城市生活更方便、 更安宁、更舒心！

（八）扎扎实实推进重点领域改革创新。紧跟中央、省改革步伐，结合我市实际落地各项改革措施。

创新投融资机制。 全面开放市场， 大力引进各类金融机构， 加强与国有政策性银行的合作， 搭建资本平台。统筹利用银行、 保险、 担保、 基金、 投资公司、 小额贷款等多种金融资源， 充分发挥国有企业、 国有合资企业的融资平台作用。 积极利用国家级贫困县 （区） 企业上市绿色通道政策， 内培外引， 年内增加拟上市企业60家以上。 盘活政府经营性资产， 加快企业优化重组， 大力推进企业资产证券化。 积极发展混合所有制， 推进国有企业薪酬制度改革。 推行政府和社会资本合作、 融资租赁、 金融租赁、 专项基金等筹资方式， 加快基本建设，推动产业发展。 支持组建张家口民营投资集团， 在激发社会资本活力方面起到带动作用。

创新政府行政方式。 优化营商环境， 强力推进 “放管服”改革。以清单管理推动简政放权。市级承接和保留的行政审批事项再取消或下放四分之一以上。 推进企业注册登记、注销便利化，清理规范行政审批中介服务和收费。大力简化审批手续，优化审批流程，推动行政审批标准化。完成市、县 （区）两级行政审批局组建。加强事先指导和事中事后监管，做到 “双随机、一公开”监管全覆盖。推进政府预决算公开， “三公”经费再压缩5%，坚守节用裕民， 挤出更多资金改善民生。

创新开发区体制机制。全面引入市场机制，深化机构编制、人事薪酬、行政审批、投融资平台建设四项重点改革，全面实行聘任制和绩效工资，理顺园区与县区的关系。明确岗位职责，强化效能考核。健全激励机制，提高招商引资和项目建设效率。对接优秀企业，引进优质项目，打造骨干企业为龙头、上下游配套的产业集群，建设具有鲜明产业特色的专业园区，使开发区真正成为聚集产业、推动发展的主战场。今年，经济技术开发区要争创国家级开发区。

（九）扎扎实实推进以保障和改善民生为重点的社会建设。 坚持民生优先， 切实保障基本， 尽心竭力服务人民群众。

大力做好就业再就业工作。 重视高就业的实体经济发展和项目建设。 强化对灵活就业、 新就业业态的支持。 完善提升就业信息平台， 加强职业技术培训， 增强就业的精准性。 重视高校毕业生就业， 支持大学生创业， 鼓励新生代农民工进城落户就业和返乡创业。 扎实做好退役军人安置工作。 妥善做好去产能企业职工安置和转岗再就业工作。 加大就业援助力度， 扶持城镇困难人员、残疾人就业， 确保零就业家庭至少有1人稳定就业。年内实现城镇新增就业7万人。 通过扎扎实实的努力， 让张家口人更多在家门口就业。

大力办好公平优质教育。依据城乡规划，适应区划调整和新型城镇化及时优化教育布局。推进城乡教育一体化均衡发展，主城区和县城开展六年一贯制试点，实施集团化、联盟化办学，着力解决 “大班额” “入学难”“择校热” 问题。 优化农村中小学校布局， 采取农村教师“去校籍”管理模式，均衡配置教师资源。启动普惠幼儿园集团化建设工程，年内全市新建50所普惠制幼儿园。新建、改建438所中小学校。 深化与知名企业合作， 发展面向市场、服务发展、促进就业的职业教育。建立教育奖励基金，推动名师工程和中小学质量提升工程的实施， 打造品牌名校。推进 “智慧校园”建设。积极引进从幼儿园到高中阶段的国际教育学校。争取5所以上北京或其它城市优质学校来我市设立分校。

大力完善医疗养老体系。积极推进城市公立医院改革。推动医疗联合体建设，每家三级医院与3家以上县级医院联动，选派优秀骨干医生轮流到县级医院工作，方便群众就近就医。推进分级诊疗体系建设。实施家庭医生签约服务，同步建立全民电子健康档案，打造 “智慧医疗”。今年家庭医生签约服务重点人群覆盖率达到80%以上，规范建档率达到75%以上。 推进市第二医院创伤诊疗中心、120急救中心、中心血站采供血业务楼建设。大力支持社会资本进入养老领域，鼓励医养结合，积极发展农村互助养老院、居家社区养老。年内新建农村互助养老院100个。

大力提升文化体育事业。加大文物古迹和非物质文化遗产传承保护力度，打造城市名片，提升泥河湾、大境门、黄帝城、暖泉古镇、万全卫城等保护利用水平。启动堡子里整体保护开发，实施 “蔚县古堡拯救行动”。做好张库大道申遗工作，提高我市历史文化的影响力。丰富民俗社火活动，举办戏曲、曲艺演出，推动优秀戏曲进校园。市图书馆、博物馆新馆建成开放。推进体育服务体系建设，修补城市社区健身场地短板。构建市、县、乡、村四级全民健身设施网络和城市社区10分钟健身圈。

大力维护社会安全稳定。推进国家安全发展示范城市和标本兼治遏制重特大安全生产事故试点城市创建。完善安全生产责任 “一张网”，严格落实 “党政同责、一岗双责、失职追责”安全生产责任制。对重点行业和重点领域进行无死角、无缝隙监管，提高安全监管科学化规范化水平，坚决遏制重特大事故发生。以创建国家食品安全示范城市为载体，加强重点领域、重点环节的持续整治，建立质量追溯体系，确保食品安全。加强基层建设，全面推行网格化管理。加强社会治安综合治理，严厉打击各种违法犯罪，严密防范暴力恐怖事件。创新社会治理，依法管理宗教事务，完善社会矛盾排查化解机制，保持社会和谐稳定。

（十）扎扎实实加强政府自身建设。强化 “四个意识”，坚决维护以习近平同志为核心的党中央权威，自觉在思想上政治上行动上同党中央保持高度一致， 深入开展 “两学一做”学习教育，以战斗精神打造法治政府、廉洁政府、实干政府、服务政府。

要依法全面履职。自觉遵照法治精神，深入推进依法行政，把政府全部工作纳入法治框架内，在法治轨道上行使各项权力，努力打造履职到位、运转高效的政府。推进行政执法 “三项制度”改革。依法接受市人大及其常委会的监督，自觉接受市政协的民主监督，主动接受社会和舆论监督，认真听取人大代表、政协委员、民主党派、工商联、无党派人士和各人民团体的意见。围绕市委工作大局，主动加强与群团组织、社会组织的常态互动，让多方积极性竞相迸发、互济共进！

要保持廉洁行政。认真贯彻全面从严治党要求，严格落实中央 “八项规定”精神，一以贯之反对 “四风”。自觉做到依法用权、正确用权、干净用权，守纪律、讲规矩。加强行政监察和审计监督。聚焦重点领域，严查侵害群众利益的不正之风和腐败问题，注重查处 “微腐败”。

要勇于担当实干。扎实开展机关作风整顿，坚持 “八破八立”，大力解放思想，摒弃墨守成规、安于现状的等靠思想，大胆改革，锐意创新，进一步提高解决问题和推进工作的能力和水平。大力提高政府系统执行力，严格落实工作责任制，层层传导压力，确保政令畅通。严厉整肃庸政懒政怠政行为。健全激励机制和容错纠错机制，为干事者鼓劲，为担当者撑腰，让想干事、敢干事、会干事的氛围越来越浓，通过勤勉实干创造张家口美好的未来！

要延伸服务手臂。 结合深化 “放管服” 改革， 在简化优化办事审批程序的基础上， 按照 “互联网+政务服务”的运行模式， 在企业、 在社区、 在农村， 完善、 拓展、 提升既有工作网络、 工作平台。 建立为企为民办事专业队伍， 健全为企为民办事机制， 提高为企为民办事效率， 全面推进 “网上政府” 网上办、 “身边政府” 身边办， 以实实在在心贴心的服务践行党的宗旨， 密切党群干群关系，赢得各方支持， 凝聚起万众一心、 众志成城促发展的磅礴力量！

各位代表！两周前，中国共产党张家口市第十一次代表大会刚刚胜利闭幕。抢抓京津冀协同发展、京张携手筹办冬奥会、建设可再生能源示范区重大历史机遇，加快转型、绿色发展、跨越提升，完成三大历史任务、交出两份优异答卷的大考试已经开启。让我们更加紧密地团结在以习近平同志为核心的党中央周围，在省委、省政府和市委的坚强领导下，齐心协力，团结奋斗，夙兴夜寐，激情工作，以优异成绩迎接党的十九大胜利召开！
